# A Single High-Intensity Shock Wave Pulse With Microbubbles Opens the Blood-Brain Barrier in Rats

**DOI:** 10.3389/fbioe.2020.00402

**Published:** 2020-05-05

**Authors:** Yi Kung, Hsin-Yu Huang, Wei-Hao Liao, Abel P.-H. Huang, Ming-Yen Hsiao, Chueh-Hung Wu, Hao-Li Liu, Claude Inserra, Wen-Shiang Chen

**Affiliations:** ^1^Department of Physical Medicine and Rehabilitation, National Taiwan University Hospital, National Taiwan University College of Medicine, Taipei, Taiwan; ^2^Department of Surgery, National Taiwan University Hospital, Taipei, Taiwan; ^3^Department of Electrical Engineering, Chang Gung University, Taoyuan, Taiwan; ^4^INSERM, U1032, LabTAU, Universiteì Claude Bernard Lyon 1, Lyon, France

**Keywords:** high-intensity pulsed ultrasound, blood-brain barrier, microbubbles, cavitation, safe dose

## Abstract

Focused extracorporeal shockwave (FSW), one kind of focused high-intensity pulsed ultrasound, has been shown to induce blood-brain barrier (BBB) opening in targeted brain areas in rat animal models with minimal detrimental effects below threshold intensity levels or iterations. In the current study, we found that the thresholds could be further reduced by the addition of microbubbles (ultrasound contrast agents or UCA; SonoVue). FSW with 2 × 10^6^ MBs/kg of UCA (20% of clinical dosage) at an intensity level of 0.1 (peak positive pressure 5.4 MPa; peak negative pressure −4.2 MPa; energy flux density 0.03 mJ/mm^2^) resulting in a 100% BBB opening rate without detectable hemorrhage or apoptosis in the brain. Significantly reduced free radical production was found compared with 0.5 MHz focused ultrasound at a peak negative pressure of 0.44 MPa (1% duty cycle and 4 × 10^7^ MBs/kg of UCA). FSW devices offer advantages of commercial availability and high safety, and thus may facilitate future research and applications of focal BBB opening for oncological and pharmacological purposes.

## Introduction

The blood brain barrier (BBB) is a multicellular vascular structure which controls the passage of molecules and ions between the bloodstream and the brain. It sustains an environment that assures synaptic transmission and neuronal function by selectively restraining the diffusion of hydrophilic molecules and pathogens from entering the brain parenchyma. Nevertheless, it also prevents the diffusion of large-molecule neuropeptides and around 99% of small molecules drugs from entering the brain, and thus poses a major obstacle for medical treatment of CNS related diseases (Pardridge, 2005; Tominaga et al., 2016; Cho et al., 2017).

Four strategies have been developed to overcome the drug delivery challenge posed by the BBB: (i) intra-arterial infusion of hyperosmolar solutions; (ii) direct invasive injection of vasoactive drugs into the target area to bypass the BBB; (iii) encapsulating drugs into nanoparticles for delivery across the BBB; and (iv) by injecting ultrasound contrast agent (UCA) intravenously and simultaneously applying focused high-intensity pulsed ultrasound (HIPU) to open the BBB in targeted brain areas (Fan et al., 2014b; d’Angelo et al., 2019). The first strategy induces a transient rise in intracranial pressure, causes non-selective opening of the BBB in the vascular territory, and exposes large volumes of brain tissue to potentially toxic substances (Bellavance et al., 2008). The second strategy requires invasive procedures and is limited by parenchymal drug diffusion. The efficacy of the third strategy is limited by nanoparticle toxicity and bio-distribution (De Jong and Borm, 2008; Raimondi et al., 2020). The fourth strategy, HIPU-UCA, was found induce transient tissue edema, neuronal function suppression (Chu et al., 2015), astroglial scarring (Kobus et al., 2016), transient ischemia, intracerebral hemorrhage (Fan et al., 2012), and sterile inflammation (McMahon et al., 2017; Tharkar et al., 2019) in addition to BBB opening.

Our previous study showed that BBB opening can be precisely controlled in terms of depth, size and location by a focused extracorporeal shockwave (FSW) device (Kung et al., 2018), originally designed for the treatment of various soft tissue pathologies (Sethu et al., 2015; Reilly et al., 2018). FSW operates at a lower frequency than HIPU, producing less transcranial attenuation and better penetration. Suitable FSW devices are commercially available and thus no complicated HIPU devices are needed for CNS applications. Similar to HIPU, FSW is well known to produce cavitation (Bachmann et al., 2001), which is believed to be the major mechanism responsible for BBB opening caused by negative pressure-induced cavitation (Wu, 2016; Hsu et al., 2018). However, BBB opening requires high FSW pressure levels required, and its safety has yet to be clarified. This study aims to develop strategies to improve the safety margins of FSW in brain applications.

## Materials and Methods

### Bio- and Chemical Materials

The study proposal was approved by the ethics committee of the Laboratory Animal Center at the National Taiwan University College of Medicine (approval No. 20170091 for the use of rats), and adhered to the experimental animal care guidelines. All rats (adult Sprague Dawley rats between 9 and 10 weeks of age) were obtained from the National Laboratory Animal Center (Taipei, Taiwan), and were divided into two groups, receiving FSW with (20 rats) and without (15 rats) the infusion of microbubbles. Another 15 rats were used to evaluate the duration of FSW-UCA induced BBB opening. One hundred fifty millimolars NaCl sterile-filtered by 0.22 μm PES membrane (Millipore syringe filter) was acquired from Polyplus-transfection (Illkirch, France). Terephthalic acid (TA), Cytochrome C (CytoC), Tris base, Hydrochloric acid, Sodium phosphate dibasic, and Potassium dihydrogen phosphate were purchased from Sigma-Aldrich, Inc. (Missouri, United States). Singlet Oxygen Sensor Green (SOSG) was obtained from Thermo Fisher Scientific Inc. (Waltham, MA, United States). Forane (Isoflurane) was acquired from Aesica Queenborough Ltd. (Queenborough, United Kingdom). Isotonic sodium chloride solution (0.9%) was provided by Taiwan Biotech Co., LTD. (Taoyuan, Taiwan). A peroxidase *in situ* apoptosis detection kit (TUNEL S7100, ApopTag) was purchased from Merck KGaA (Darmstadt, Germany). SonoVue (UCA) was acquired from Diagnostics Inc. (Milan, Italy). Ultrasound coupling gel (CG955, sonic resistance: 1.55 ± 0.05 MRayl, pH 7.0 ± 0.05) was obtained from Ceyotek (Chiayi City, Taiwan).

### Instruments and Devices

The FSW device (PiezoWave) was purchased from Richard Wolf GmbH (Knittlingen, Germany). A 500 kHz × 64 mm focused piezoelectric transducer (H-104G) and its fundamental and third harmonic resonance impedance matching network were acquired from Sonic concepts, Inc. (Washington, United States). A RF Power Amplifier (40AD1) was obtained from Amplifier Research Inc. (Pennsylvania, United States). A RF multifunction power meter (4421) and its directional power sensor (4025) were procured from Bird Technologies Co. (Ohio, United States). A function/arbitrary waveform generator (33120A) was purchased from Agilent Technologies, Inc. (California, United States). An oscilloscope (LT354ML) was obtained from LeCroy Co. (New York, United States). An immersion planar ultrasound transducer (1 MHz, A392S-SU) and manually controlled ultrasound pulser-receivers (5072PR) were acquired from Olympus Co. (Tokyo, Japan). A slide scanner (Ventana Dp200) and its software (Ventana Image Viewer v3.2) were obtained from F. Hoffmann-La Roche Ltd. (Basel, Switzerland). A microplate reader (Infinite 2000 Pro) and its software (i-control) were procured from Tecan Austria GmbH Co. (Grodig, Austria).

### Transcranial Attenuation of Focused Shock Wave (FSW)

**FIGURE 1:**
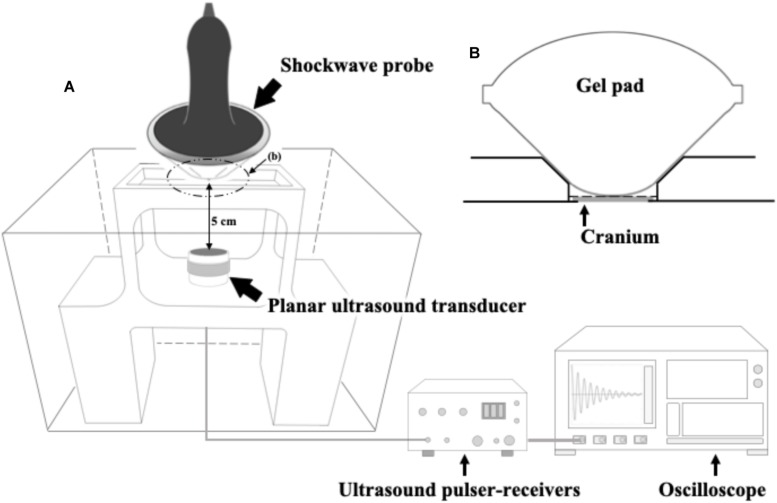
**(A)** The ultrasound receiver-based transcranial attenuation system for FSW and its experimental setup; **(B)** experimental implementation of the FSW probe and a rat cranium, wherein the gray block is an isolated rat cranium (around 0.75 mm), or other tested materials, i.e., a 0.75 mm of polystyrene plate (PP) or a 0.75 mm of BIO-RAD adhesive sealer (AS). In which, the depth from the gel pad bottom to the focus of the FSW probe is 5 mm. The distance between the gel pad and the immersed planar ultrasound transducer is 5 cm as shown on the **(A)**. **(B)** shows the zoomed view of the dotted cycle, **(B)**, on **(A)**. The gel pad bottom is in close contact with the rat cranium on the **(B)**.

Figure 1A shows the setup of the ultrasound receiver-based transcranial attenuation system for measuring the relative pressure levels of our FSW transducer. The concave FSW probe (radius of 46 mm and curvature radius of 62.9 mm) was coupled with a gel pad to ensure the focus of the FSW probe was 5 mm from the bottom of the gel pad (as shown in Figure 1B). The FSW, together with the gel pad, was positioned on the center top of the isolated rat cranium to ensure a 5 cm distance between the cranium surface and the immersed planar ultrasound transducer (38.1 mm in diameter). A 10-times distance difference between FSW focal length (5 mm) and attenuation measuring length (5 cm) was used to prevent damage to the immersed planar ultrasound transducer by the high intensity FSW pulses.

### FSW Induced BBB Opening and the Effect of UCA

To evaluate the performance of FSW-induced BBB opening with or without the presence of microbubbles, the following procedure was modified based on previous studies (Chu et al., 2013; Kung et al., 2018). The intensity levels for each parameter in this research are shown in Table 1 (calibration data obtained from Richard Wolf GmbH).

**TABLE 1:** Major FSW intensity levels and the corresponding acoustic pressures.

Intensity level	0.1	15	17	20
Peak negative pressure (MPa)	−4.20	−15.80	−16.96	−18.70
Peak positive pressure (MPa)	5.40	53.10	62.94	77.70
Energy flux density (mJ/mm^2^)	0.03	0.59	0.68	0.82

To evaluate the performance of FSW applications, the successful (visible) BBB opening rate is defined as number of rats with visible Evans blue (EB) leakage after FSW treatment over the total number of rats receiving FSW treatment with a cut-off value. To define the cut-off value, the positive EB-stained area of the histology sections was analyzed using a color histogram of Image J based on the differences in number of blue pixels between the FSW-applied side and the untreated side.

UCA has been widely used with previous HIPU studies to induce BBB opening in the brain by enhancing cavitation (Yang et al., 2011; Chu et al., 2013), thus the effect of UCA on FSW was investigated by infusing various concentrations of SonoVue UCA from 2.5 × 10^2^ to 2.5 × 10^8^ microbubbles/kg (MBs/kg) body weight (the clinical concentration is 3 – 15 × 10^6^ MBs/kg) through the tail vein immediately before FSW treatment (*N* = 5).

Moreover, to evaluation the duration of FSW-UCA induced BBB opening, 15 rats were treated with FSW at time 0. 0.5 ml of 3% EB were infused immediately before sacrificing the rats for brain sectioning 1, 2, and 3 h (five rats in each time point) after time 0.

### Quantification of FSW and HIPU-Induced Free Radical Generation

Cavitation is believed to be the major mechanism responsible for BBB opening by FSW and HIPU, and also produces free radicals during treatment (Bachmann et al., 2001; Wu, 2016; Hsu et al., 2018). Excessive free radical generation, especially reactive oxygen species (ROS) and reactive nitrogen species (RNS) leads to radical stress in the biological system, and has been implicated in pathogenesis and pathological conditions associated with apoptosis and inflammation (Fubini, 2003; Fan et al., 2014a). Therefore, an adequate understanding of the free radical stress-associated phenomenon could underpin the development of targeted FSW and HIPU-based therapeutic interventions.

For this reason, a terephthalate dosimeter, singlet oxygen sensor green, and Cytochrome C were used to investigate cavitation-induced free radical generation during FSW and HIPU-induced BBB opening. The terephthalate dosimeter and singlet oxygen sensor green respectively react with the hydroxyl radicals and singlet oxygen (^1^O_2_) to generate hydroxyterephthalic acid (HTA, ex 323 nm/em 424 nm) and SOSG endoperoxides (ex 488 nm/em 525 nm) (Mason et al., 1994; Iida et al., 2005; Circu and Aw, 2010; Harad et al., 2013; Lo et al., 2014). The cytochrome C (abs 550 nm) is reduced with nitric oxide, superoxide, hydrogen peroxide, peroxynitrite, and nitrogen dioxide (Hill et al., 1996; Kondo et al., 1996; McEwan et al., 2010).

Three conditions which induce cavitation were studied: FSW-UCA, HIPU-UCA, and FSW alone. The HIPU-UCA group was processed using a previously employed protocol, i.e., 0.5 MHz HIPU, 0.62 mechanical index, 1% duty cycle, 1 Hz pulse repetition frequency (PRF), 60 s in duration, and UCA at the concentration of 4 × 10^7^ MBs/kg (four times of clinical dose or CD) (Barteri et al., 1996). The parameters used for the FSW-UCA group are intensity level 0.1 (peak positive pressure 5.4 MPa; peak negative pressure −4.2 MPa; energy flux density 0.03 mJ/mm^2^), single pulse, UCA 2 × 10^6^ MBs/kg (1/5 CD), while the FSW alone group has an intensity level of 0.1, and a single pulse.

Prior to use, three ROS indicators were prepared as 2 mM TA in 50 mM PBS (pH7.3 ± 0.1), 1 μM SOSG in 50 mM Tris (pH 7.0 ± 0.1), and 50 μM cytochrome C in 10 mM Tris (pH7.0 ± 0.1) solutions following the above cited studies. Then, 380 μl of the ROS indicator solution in a 96-well plate was placed above a FSW or HIPU probe. After exposure, aliquots (200 μl) of the solution were transferred to other 96-well plates and ROS measurements were conducted using the microplate spectrophotometer system.

### Histopathologic Sections

To evaluate the extent of tissue damage caused by various FSW intensities, durations and UCA concentrations, rat brains were sliced using brain matrices following the formaldehyde fixed process. After sacrificing, the brains were then immersed in 10% formaldehyde solution for 24 h. Subsequently, the sliced specimens were embedded in paraffin and subjected to H&E (for hemorrhage), TUNEL assay (for apoptosis), or GFAP stain (for glial cells). Slides were analyzed using a Ventana Dp200 slide scanner with its software, Ventana Image Viewer v3.2 advanced.

### Statistics

All data are expressed as mean ± standard deviation (SD) of at least five independent samples (N). In group comparisons, all statistical evaluations were carried out with one-way ANOVA and *post hoc* analysis (Tukey). A p-value of less than 0.05 was considered significant.

## Results

### Transcranial Attenuation of FSW

**FIGURE 2:**
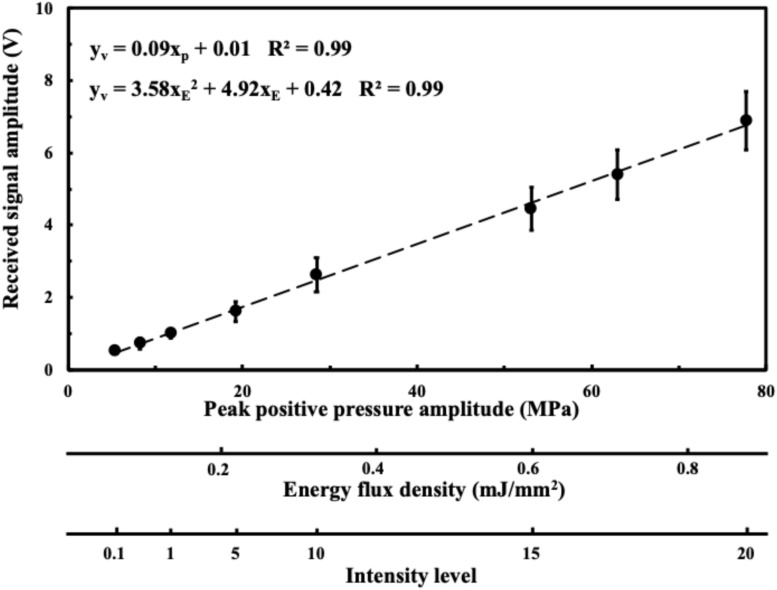
Received acoustic amplitude in a peak positive pressure domain; in an energy flux density domain; in an intensity domain. Data are reported as average (dots) and standard deviation (error bars) of 100 pulses.

Figure 2 shows the acoustic absorption response of the FSW in a peak positive pressure domain and an energy flux density domain. From Figure 2, it can be seen that the FSW output is widely distributed with a large ± SD.

**FIGURE 3:**
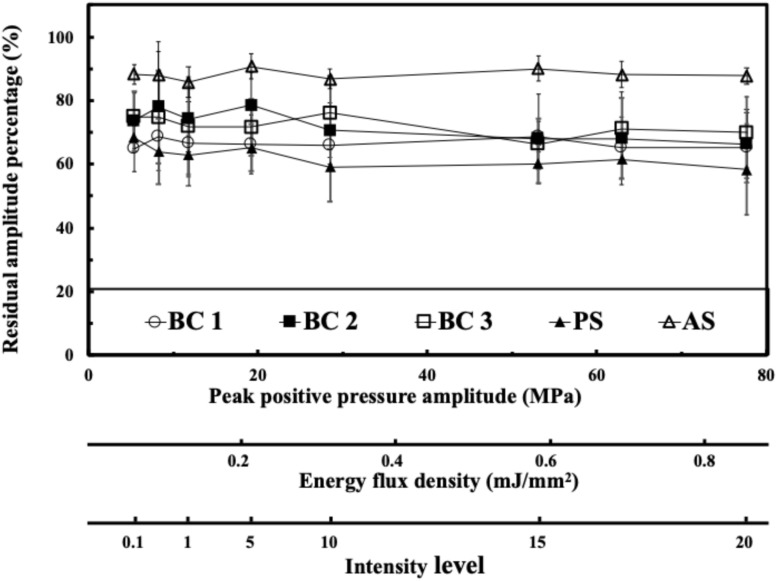
Transcranial attenuation of focused FSW in an energy flux density domain; in an intensity domain with different material, in which BC is the braincase (around 0.75 mm, 3 cases), PS is the polystyrene plate (0.75 mm), and AS is the adhesive sealer (0.75 mm). Data are reported as average (dots) and standard deviation (error bars) of 100 pulses.

Figure 3 shows the residual acoustic pressure response of the FSW to a peak positive pressure domain and an energy flux density domain for different media. After the FSW passed through a 0.75 mm of adhesive sealer, 0.75 mm of braincase, or a 0.75 mm of polystyrene plate, the remaining acoustic pressure amplitude was respectively about 85, 70–75, and 60%.

### Threshold and Opening Duration of FSW Induced BBB Opening With UCA

**FIGURE 4:**
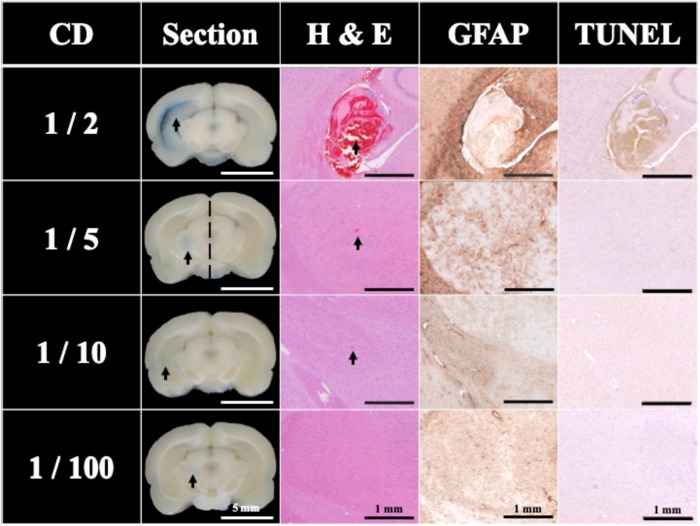
Histology sections of H&E, TUNEL, and GFAP stains for a single FSW pulse under intensity level 0.1 (peak positive pressure 5.4 MPa; peak negative pressure −4.2 MPa; energy flux density 0.03 mJ/mm^2^) with different concentrations of UCA (SonoVue), where CD is the clinical dosage (10^7^ MBs/kg). *N* = 5. The left side brain on the 1/5 CD (2 × 10^6^ MBs/kg of UCA) group was defined as the cut-off value of BBB opening blue pixels in the RGB-image vs. right side brain. The indicator was 3% of EB (pre-dissolved in 0.9% saline). The scale bar was 5 mm on sections, and 1 mm on H&E, TUNEL, and GFAP stains. MBs, microbubbles.

Traditionally, UCA is used with HIPU to induce BBB opening. Figure 4 shows the effect of different concentrations of UCA on BBB opening using FSW (*N* = 5). The BBB opening region (the blue-stained area indicated by the arrows) could be repeatedly produced at the junction of the cortex and subcortical area of the rat brain. In this study, the cut-off value threshold of successful BBB opening was defined by the difference (around 5,000) of integral blue pixels in the RGB-image between the left half brain (FSW treated side) and the right half brain (FSW untreated side) for the 1/5 CD group in Figure 4. Based on the cut-off value threshold of successful BBB opening, the successful ratios of the 1/2 CD, 1/5 CD, 1/10 CD, and 1/100 CD group in Figure 4 are, respectively, 100, 100, 40, and 20%, where CD is 10^7^ MBs/kg. As a consequence, the BBB opening is accompanied by red blood cell extravasations, as shown in the 1/5 CD group H&E stain (arrowed) in Figure 4. Unfortunately, visible bleeding and vessel breakage occur during the FSW-UCA process, as shown by the 1/2 CD group H&E stain. The probability of visible bleeding and vessel breakage of the 1/2 CD and 1/5 CD group in Figure 4 are, respectively, 80 and 20%.

Figure 4 also compares the H&E stain, TUNEL, and GFAP stain indicating that the 1/5 and 1/10 CD groups showed around 0.0001 mm^2^ red blood cell extravasation in the H&E stain (arrowed). Light cell apoptosis is shown by the concentrated cell nucleus (dark brown particles) on the TUNEL assay, and prosperous astrogliosis is shown by rich astrocytes (brown star-shaped cells) on the GFAP stain. On the other hand, RBC extravasation was absent in the 1/100 CD group. Therefore, this condition (intensity level 0.1, 1 pulse; peak positive pressure 5.4 MPa; peak negative pressure −4.2 MPa; energy flux density 0.03 mJ/mm^2^), 1/5 CD (2 × 10^6^ MBs/kg) was selected as the threshold to induce 100% successful BBB opening using FSW with 1/5 CD of UCA.

**FIGURE 5:**
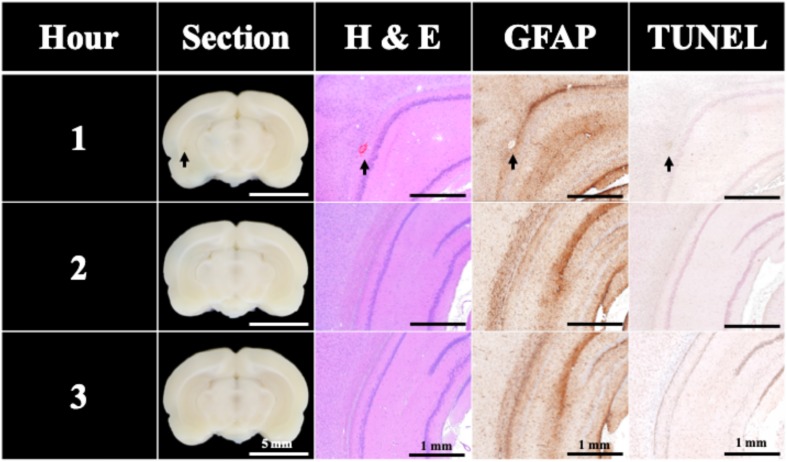
BBB opening was found only 1 h after a single FSW pulse under intensity level 0.1 (peak positive pressure 5.4 MPa; peak negative pressure −4.2 MPa; energy flux density 0.03 mJ/mm^2^) with 1/5 CD of UCA (2 × 10^6^ MBs/kg; SonoVue). *N* = 5. The indicator was 3% of EB (pre-dissolved in 0.9% saline). The scale bar was 5 mm on sections, and 1 mm on H&E, TUNEL, and GFAP stains. MBs, microbubbles.

To evaluate the BBB opening duration after FSW treatments, EB was used as an indicator to show the BBB opening in rat brains since EB cannot penetrate an intact BBB. In this study, EB can only be found in the 1 h group in Figure 5. Only 40% of brain sections in the 1 h group exceed the BBB opening threshold, thus the BBB opening duration of a single-shot FSW treatment is around 1 h.

Only two instances of slight visible bleeding and vessel breakage can be found in the H&E stains of the 1 h group in Figure 5; other H&E, TUNEL, and GFAP stains shows no significant difference.

Based on the results of Figures 4, 5, under the condition of single pulse under intensity level 0.1 (peak positive pressure 5.4 MPa; peak negative pressure −4.2 MPa; energy flux density 0.03 mJ/mm^2^) with 1/5 CD of UCA (2 × 10^6^ MBs/kg of UCA; SonoVue), there is only a 0.15% chance of very slight visible bleeding or vessel breakage.

### Threshold of FSW Induced BBB Opening Without UCA

**FIGURE 6:**
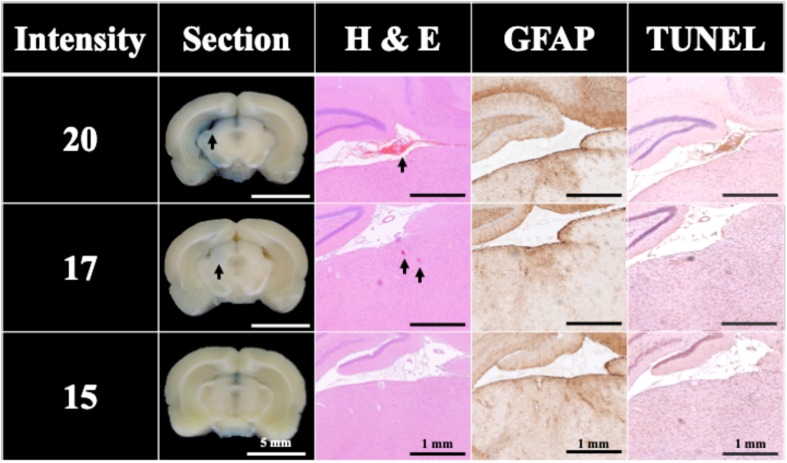
Histology sections of H&E, TUNEL, and GFAP stains for a single FSW pulse at intensity level 20 (peak positive pressure 77.70 MPa; peak negative pressure −18.70 MPa; energy flux density 0.82 mJ/mm^2^), intensity level 17 (peak positive pressure 62.94 MPa; peak negative pressure -16.96 MPa; energy flux density 0.68 mJ/mm^2^) and intensity level 15 (peak positive pressure 53.10 MPa; peak negative pressure −15.80 MPa; energy flux density 0.59 mJ/mm^2^). *N* = 5. The indicator was 3% of EB (pre-dissolved in 0.9% saline). Scale bar was 5 mm on sections, and 1 mm on H&E, TUNEL, and GFAP stains.

Figure 6 shows that the BBB could still be successfully opened without UCA using only a single pulse at intensity levels higher than 17. But the successful opening rate dropped to 60% (at intensity level 20; peak positive pressure 77.70 MPa; peak negative pressure −18.70 MPa; energy flux density 0.82 mJ/mm^2^) with visible bleeding and 20% (at intensity level 17; peak positive pressure 62.94 MPa; peak negative pressure -16.96 MPa; energy flux density 0.68 mJ/mm^2^) without visible bleeding, whereas the opening rate at intensity levels under 15 (peak positive pressure 53.10 MPa; peak negative pressure −15.80 MPa; energy flux density 0.59 mJ/mm^2^) was 0%. Figure 6 also compares the H&E stain, TUNEL, and GFAP stain, where the intensity level 20 and 17 groups showed respectively vessel breakage (arrowed on Intensity 20) and around 0.0002 mm^2^ red blood cell extravasation (arrowed on Intensity 17) in the H&E stain. Light cell apoptosis was shown by the concentrated cell nucleus on the TUNEL assay, and prosperous astrogliosis was shown by rich astrocytes on the GFAP stain. On the other hand, RBC extravasation was absent in the intensity 15 group. Therefore, a single FSW pulse at intensity level 20 (peak positive pressure 77.70 MPa; peak negative pressure −18.70 MPa; energy flux density 0.82 mJ/mm^2^) was selected as the threshold to induce 60% BBB opening without the addition of UCA.

### Comparison of FSW and HIPU Induced Free Radical Generation

**FIGURE 7:**
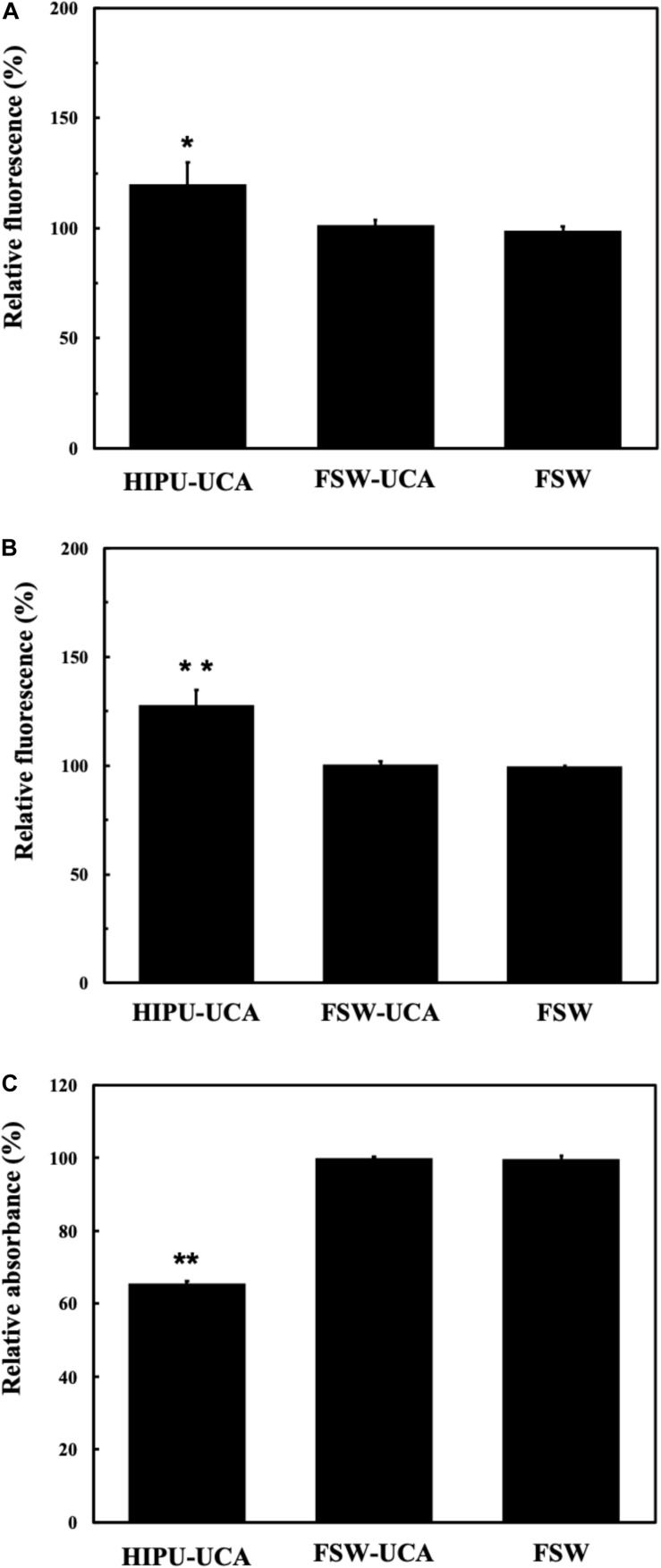
**(A)** Relationship of the relative fluorescence levels of TA between untreated control group (as 100%) and groups treated by the BBB opening condition of HIPU-UCA, FSW-UCA, and FSW. **(B)** Relationship of the relative fluorescence levels of SOSG between untreated control group (as 100%) and treated by the BBB opening condition of HIPU-UCA, FSW-UCA, and FSW groups. **(C)** Relationship of the relative absorbance levels of Cytochrome C between untreated control group (as 100%) and groups treated by the BBB opening condition of HIPU-UCA, FSW-UCA, and FSW. *N* = 8. *p < 0.05 vs. control in the *t*-test. **p < 0.01 vs. control in the *t*-test.

As shown in Figures 7A–C, only the HIPU-UCA group demonstrates significant free radical generation for each indicator (TA, SOSG, Cytochrome C) under BBB opening conditions.

## Discussion

This study compares and characterizes the efficiency of FSW induced BBB opening with or without the use of UCA. Both FSW-UCA and FSW alone produced BBB opening, and the quantitated degrees of surrogate molecule penetration were compared. The results imply that by controlling FSW-UCA and FSW dose administration, the BBB-opening effect can be predicted.

**FIGURE 8:**
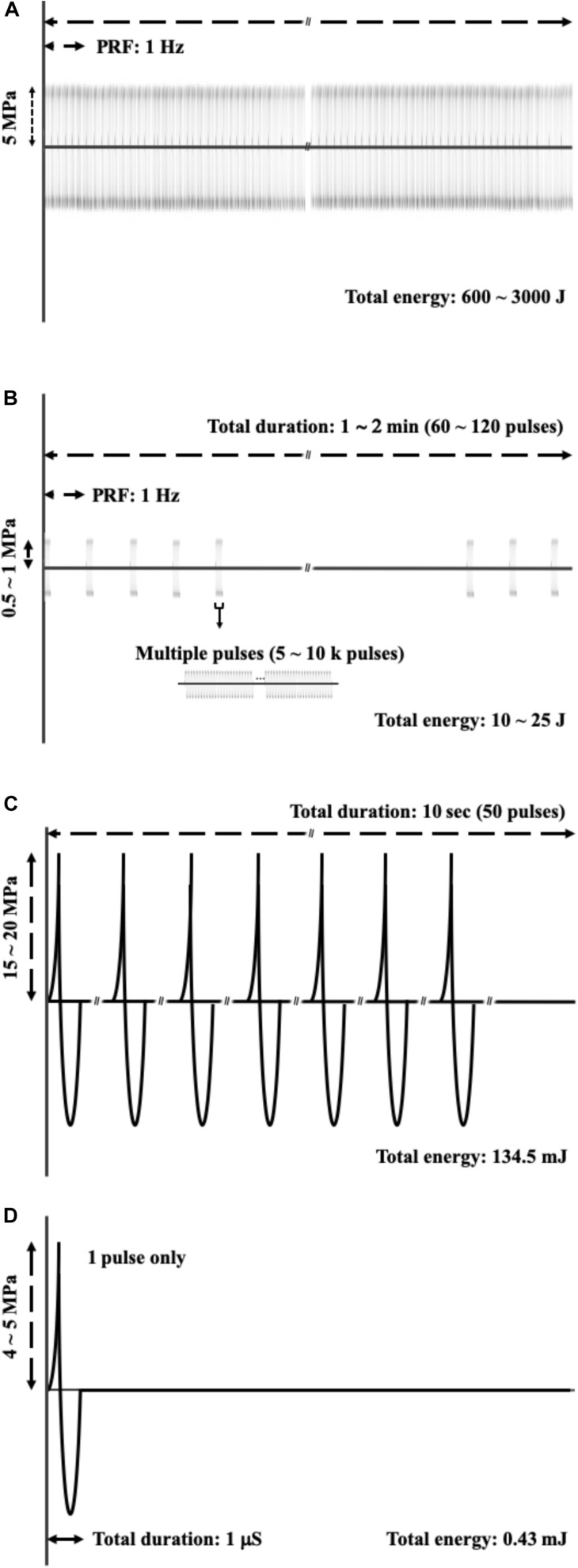
Comparisons of acoustic profile of **(A)** HIFU applied on the tumor ablation; **(B)** HIPU, the preferred method to induce BBB opening; **(C)** burst FSW (our previous study), which is also applied for BBB opening; **(D)** a single shot FSW, which is the method used in this study.

Figure 8 shows the physical characteristics of various therapeutic acoustic devices, including the high-intensity focused ultrasound (HIFU) device for tumor ablation (Figure 8A), HIPU, a preferred device to induce BBB opening (Figure 8B), burst FSW [our previous study (Kung et al., 2018)] for BBB opening (Figure 8C), and single-shot FSW (Figure 8D), the device used in the current study. The total energy required for BBB opening under the conditions shown in Figure 8 were also compared, with that in Figure 8D, respectively, about 1/60,000 times that of the conditions in Figure 8B and 1/300 times that of the conditions in Figure 8C. In addition, the energy in Figure 8D is only 1/7,000,000 of that in Figure 8A. The major improvement between this study (a single-shot FSW) and our previous study (burst FSW) is the use of UCA for inducing BBB opening, which not only successfully reduces the required energy and pulses, but also provides a UCA dose guide for future related single-shot FSW research with drugs containing UCA.

On the other hand, as shown in Figures 8B,D, the HIPU and a single-shot FSW have four major differences. The first difference is the numbers of pulses, with the single-shot FSW emitting one pulse only, while HIPU has 300–12,000 k pulses. The second difference is the acoustic pressure, which for the single-shot FSW is around −4 to −5 MPa (peak-negative pressure), but HIPU is −0.5 to −1 MPa. The third difference is the total duration, which for the single-shot FSW is around 1 ms, but for the HIPU is as long as 60–120 s. The last difference is the mechanical wave profile, in which FSW has a very sharp positive pulse as shown in the upper part of Figure 8C, while HIPU has regular sine waves (Bachmann et al., 2001; Kung et al., 2018; Huang et al., 2019).

As shown in Figure 2, the distributions of received acoustic signal amplitudes varied, probably due either to the jitter in the amplitude of the pulse emitted from the FSW device, or to the presence of a cavitation liquid consisting of a superposition of the pressure induced by a direct shockwave and from secondary waves emitted by collapsing bubbles, wherein the spatial randomness of the secondary waves often leads to the noise-like signal registered (Zijlstra and Ohl, 2008). Using degassed water and adding a small amount of acetic acid may reduce this variability since cavitation can be reduced by chemically dissolving calcite particles which serve as cavitation nuclei (Eisenmenger and Pecha, 2003). Major pressure measurements are currently performed using fiber-optic hydrophones, which entail high cost and may be too fragile to withstand pressure variations and cavitation events. Moreover, if the tip of the fiber breaks, the fiber has to be cut and stripped, and the device has to be recalibrated and repositioned, which is a time-consuming process (Ueberle, 2011; Kang et al., 2014; Liu et al., 2014; Tsai et al., 2016).

After clarifying the transcranial efficiency of FSW, future clinical applications require establishing a safe threshold to minimize tissue damage during the BBB opening process. As shown in Figures 4, 6, increasing the UCA concentration or employing higher intensity FSW treatments may induce larger areas of BBB opening, but also produce tissue damage (e.g., red blood cell extravasation, apoptosis and glia cell infiltration). The extensive red blood cell extravasation produced by the addition of UCA is probably due to strong cavitation generated by the high peak negative pressure of the FSW (see Table 1 for detailed parameters) (Bachmann et al., 2001; Wu, 2016). Therefore, according to the BBB opening ratios and red blood cell extravasation levels in Figures 4–6, the threshold of FSW with UCA could be set at a single pulse of FSW with 1/5 CD (2 × 10^6^ MBs/kg), at intensity level 0.1 (peak positive pressure 5.4 MPa; peak negative pressure −4.2 MPa; energy flux density 0.03 mJ/mm^2^). For FSW without UCA, the threshold could be set at intensity level 20. At these safe thresholds, the BBB could be successfully opened with only minimal or no detectable red blood cell extravasation and a small area of inflammation observed by GFAP staining. Moreover, due to the huge difference in the intensity level of FSW (intensity 0.1 vs. 20) and the higher success rate (100% vs. 60%) with and without the infusion of UCA, UCA is preferable since a high intensity level of FSW usually implies a larger focal area, thus increasing the chance of unwanted side effects outside the targeted brain area. On the other hand, in some cases (e.g., tumor treatment) a larger focal area is needed using a higher intensity level (intensity 20). Moreover, the 1 h BBB opening duration at intensity level 0.1 (peak positive pressure 5.4 MPa; peak negative pressure −4.2 MPa; energy flux density 0.03 mJ/mm^2^) with 1/5 CD (2 × 10^6^ MBs/kg) of UCA is shorter than the 3 h in our previous research. A shorter BBB opening duration can reduce the risk of some bio-harmful substances crossing into the parenchyma and central nervous system during the opening period (Chu et al., 2013; Tominaga et al., 2016; Cho et al., 2017).

Ultrasound induced bioeffects are believed to be caused not only by mechanical effects, but also by excessive heating and free radical formation from cavitation. The excessive heating will denature tissues, and the free radicals will cause damage to cell components such as proteins, DNA, and cell membranes by oxidation (Filipczynsky and Wojcik, 1991). Fortunately, probably due to the very short pulse duration of FSW, the thermal injury to soft tissue can be ignored. Even after 3000 FSW iterations (PRF 1 Hz, P^+^ 80 MPa), the maximum temperature elevation is only 0.66°C at the focal region (Ueberle and Rad, 2011; Wang et al., 2016).

Similar conditions apply to the generation of free radicals. FSW generated significantly fewer free radicals compared with 0.5-MHz HIPU under BBB opening conditions. Excessive free radicals, especially ROS and RNS, induced radical stress and caused significant damage to cell structures, apoptosis, inflammation, and DNA degradation (Saeidnia and Abdollahi, 2013). Figure 7A shows the hydroxyl radical changes measured by terephthalate dosimeter, which is responsible for most of the oxidative damage to proteins, lipids, sugars, and nucleic acids (Fischer et al., 2013). Figure 7B shows the singlet oxygen changes measured by singlet oxygen sensor green, which not only impaired biological function, but also caused further biological damage, either through oxidization (such as lipid peroxidation products and oxidized amino acid intermediates) or as genotoxic agents (Wu et al., 1996). Figure 7C shows the changes of nitric oxide, superoxide, hydrogen peroxide, peroxynitrite, nitrogen dioxide by cytochrome C, which are related to the disruption of intracellular redox homeostasis, and irreversible oxidative modifications of lipid, protein or DNA (Circu and Aw, 2010). According to the results shown in Figures 7A–C, FSW-BBB opening produced fewer free radicals than HIPU-BBB opening, possibly due to the huge difference in on-times between BBB opening produced by HIPU-UCA (1% duty cycle, 1 s total time), FSW-UCA (only a single pulse). Due to this reduction in free radical generation, FSW-BBB opening is potentially safer due to its reduced resulting cellular apoptotic response, which may be exacerbated by intracerebral hemorrhage during BBB opening (McEwan et al., 2010; Harad et al., 2013).

Moreover, FSW-UCA provides additional benefits compared with 0.5 MHz HIPU and other HIPU devices (Table 2). In addition to reduced free radical generation, FSW-UCA requires less treatment time, provides flexible focal depth selections with gel pads, and offers easier skull penetration with lower frequency components. This study was conducted using a commercially available FSW device, thus avoiding the expense of manufacturing a dedicated HIPU device (the comparation between FSW and HIPU for BBB opening as Table 3 shown).

**TABLE 2:** Comparisons of key parameters between 0.5 MHz HIPU and FSW for opening BBB.

Device		HIPU					UCA	
		Pressure (MPa)	Frequency (MHz)	PRF (Hz)	Burst length (ms)	Duration (s)	Dose (Mega-MBs/kg)	Brand
H-104G focused ultrasound transducer (Sonic Concepts) (Wu et al., 1996)		0.44	0.50	1	10	120	40	SonoVue, Definity, USphere
Single-element FUS transducer (Imasonics SAS) (Lin et al., 2016)		0.8	0.50	1	10	120	50	SonoVue
H-107 spherical-segment FUS transducer (Sonic Concepts) (Karakatsani et al., 2017)		0.60	0.50	2	10	60	250	Self-made
H-107 spherical-segment FUS transducer (Sonic Concepts) (Samiotaki et al., 2017)		0.60	0.50	2	10	120	125	Self-made
RK100 focused ultrasound transducer (FUS Instruments Inc.) (McMahon and Hynynen, 2017)		1.09	0.55	1	10	120	200	Definity
In-house–assembled lead zirconate titanate transducer (DeL Piezo Specialties) (O’Reilly et al., 2017)		0.39	0.55	1	10	120	200	Definity

**Device**	**FSW**						**UCA**	
	**Intensity level**	**Pressure^+^ (MPa)**	**Pressure^–^ (MPa)**	**Total energy (mJ)**	**PRF (Hz)**	**Duration (s)**	**Dose (Mega-MBs/kg)**	**Brand**

Piezowave (The current study)	0.1	5.4	-4.2	0.43	1	1	2	SonoVue
	5	19.22	-9.79	134.5	5	10	–	–

**TABLE 3:** Comparison of shockwave and HIFU induced BBB opening.

	HIFU	Shockwave
Frequency range	0.5–1.0 MHz Narrow band width	Broad band with audible low frequency components
Pulse length	Short	Long
Number of waves/second	10^6^ times level	1–10 times
Energy level	10–15 J	0.43 mJ
Thermal effects	High	Low
Focus space	10 mm^3^	25 mm^3^
Free radical generation	More	Less

Based on the findings in this study, FSW induced BBB opening exhibits safe and notable therapeutic potential for the treatment of CNS diseases. The increment of endothelial permeability will allow drugs including antibiotics, biologic vehicles, chemotherapeutic agents and other medical substances with large molecular masses (>400 Dalton) to pass through the BBB, providing more options for clinical medication and achieving optimal therapeutic concentrations by enhancing bioavailability in the CNS.

## Conclusion

An ultrasound receiver-based high pulse pressure meter was developed to clarify FSW transcranial attenuation. Its relative intensity can be easily measured by an acoustic device, obviating the need for expensive optical fiber-based sensors. About 70% of the peak positive pressure of FSW is able to pass through the rat cranium, as detected by the developed device.

This study also clarifies that FSW-UCA and FSW induced BBB opening with a well-controlled exposure level is safe with acceptable levels of histopathologic change or red blood cell extravasation and apoptosis. FSW with an intravenous infusion of 2 × 10^6^ MBs/kg of UCA (20% of clinical dose) can provide safe and 100% BBB opening. A BBB opening rate of 60% can be achieved without the addition of UCA under a much higher intensity level of 20. The fine-tuned FSW based BBB opening results show simpler and improved control of cavitation-facilitated BBB opening, which may benefit future neuro-oncology and neuropharmacology research and applications.

## Data Availability Statement

All datasets generated for this study are included in the article/supplementary material.

## Ethics Statement

The animal study was reviewed and approved by the ethics committee of the Laboratory Animal Center at National Taiwan University College of Medicine (approvals No. 20170091 for the use of rats).

## Author ContrIbutions

YK and W-SC designed and developed the system and wrote the manuscript. YK and H-YH performed the major experiments. W-HL provided the biotechnological analysis information. AH, C-HW, M-YH, H-LL, CI, and W-SC directed the research.

## Conflict of Interest

The authors declare that the research was conducted in the absence of any commercial or financial relationships that could be construed as a potential conflict of interest.
